# Emergency response to terrorist attacks: results of the federal-conducted evaluation process in Germany

**DOI:** 10.1007/s00068-020-01347-8

**Published:** 2020-03-23

**Authors:** Thomas Wurmb, Axel Franke, Nora Schorscher, Barbara Kowalzik, Matthias Helm, Renate Bohnen, Jutta Helmerichs, Ulrich Grueneisen, Detlef Cwojdzinski, Georg Jung, Gesa Lücking, Martin Weber

**Affiliations:** 1 Department of Anaesthesia and Critical Care, Emergency and Disaster Relief Medicine, Head of the Subsection Emergency and Disaster Relief Medicine, University Hospital of Wurzburg, Oberdürrbacherstrasse 6, 97080 Wurzburg, Germany; 2 Department of Trauma Surgery and Orthopaedics, Reconstructive Surgery, Hand Surgery and Burn Medicine, German Armed Forces Central Hospital of Koblenz, Koblenz, Germany; 3 Department of Anaesthesia and Critical Care, University Hospital of Wurzburg, Wurzburg, Germany; 4 Head of Division Public Health Protection, Federal Office of Civil Protection and Disaster Assistance, Bonn, Germany; 5 Department of Anaesthesiology and Intensive Care Medicine, Section Emergency Medicine, Federal Armed Forces Medical Hospital, Ulm, Germany; 6 GSG 9, Federal Police Germany, Sankt Augustin, Germany; 7 Head of Division Psychosocial Crisis Management, Federal Office of Civil Protection and Disaster Assistance, Bonn, Germany; 8 President of the European Council of Disaster Medicine, Vice President of the German Society of Disaster Medicine, München, Germany; 9 Management Emergency Planning and Disaster Preparedness, Senate Department for Health, Care and Gender Equality of the City of Berlin, Berlin, Germany; 10 Medical Disaster Response Unit (MDRU), Health Protection Authority of the City of Frankfurt, Frankfurt, Germany; 11 Division 321 Health Protection, Health Security and Crisis Management of Biological Threats, Federal Ministry of Health, Bonn, Germany; 12 Program Manager Public Health Protection and Course Director, Academy for Crisis Management, Emergency Planning and Civil Protection, Federal Office of Civil Protection and Disaster Assistance, Bad Neuenahr-Ahrweiler, Germany

**Keywords:** Terror attack, Mission strategies, Lessons learned, First responders, Safety

## Abstract

**Purpose:**

Rescue missions during terrorist attacks are extremely challenging for all rescue forces (police as well as non-police forces) involved. To improve the quality and safety of the rescue missions during an active killing event, it is obligatory to adapt common rescue mission goals and strategies.

**Methods:**

After the recent attacks in Europe, the Federal Office of Civil Protection and Disaster Assistance started an evaluation process on behalf of the Federal Ministry of the Interior and the Federal Ministry of Health. This was done to identify weaknesses, lessons learned and to formulate new adapted guidelines.

**Results:**

The presented bullet point recommendations summarise the basic and most important results of the ongoing evaluation process for the Federal Republic of Germany. The safety of all the rescue forces and survival of the greatest possible number of casualties are the priority goals. Furthermore, the preservation and re-establishment of the socio-political integrity are the overarching goals of the management of active killing events. Strategic incident priorities are to stop the killing and to save as much lives as possible. The early identification and prioritised transportation of casualties with life-threatening non-controllable bleeding are major tasks and the shortest possible on-scene time is an important requirement with respect to safety issues.

**Conclusion:**

With respect to hazard prevention tactics within Germany, we attributed the highest priority impact to the bullet points. The focus of the process has now shifted to intense work about possible solutions for the identified deficits and implementation strategies of such solutions during mass killing incidents.

## Introduction

The management of terrorist attacks, rampages and other active killing events poses enormous challenges to all responding forces involved (police as well as non-police forces). After the terrorist attacks in France and Belgium in 2015 and 2016, Germany was also hit by a row of terrorist attacks. The first of these terrorist attacks took place in Wurzburg in July 2016, which produced five severely injured casualties and one fatality. The same week, a rampage in Munich killed nine people and left four injured whilst a suicide attack in Ansbach claimed several minor casualties and one fatality. The most serious attack happened in December 2016, when an islamic terrorist drove a truck into the crowd visiting a Berlin Christmas market killing 12 people and leaving 56 partly severely injured.

To evaluate the latest terrorist attacks, draw conclusions, formulate recommendations and improve those missions outcome, the German Federal Office of Civil Protection and Disaster Assistance started an evaluation process on a national level. This was done on behalf of the German Federal Ministry of the Interior and the German Federal Ministry of Health. The analysis of the terrorist attacks and the definition of the lessons learned were formulated in close collaboration with all responsible authorities, organisations and institutions directly involved in the management of active killing events.

The identified weakness, deficits and the lessons learned are presented in this paper as the basic and essential results of this evaluation process. They serve as the basis for developing new guidelines for the management of active killing events.

### Specific features on active killing events

Active killing events due to a terrorist attack or rampages have specific features, which distinguish them from any other “every day” mass casualty incidents [1–4]. One of the most important difference is that all rescue forces involved, including police, medical teams and all other rescue services, need to work hand in hand with each other at the same scene, at the same time under the highest possible time pressure constraints but with different foci [5–7].

Whilst the police have to concentrate on defusing the threat, the rescue services have to concentrate on rescuing as many casualties as possible [6, 7]. An additional difference is that the types of injuries differ profoundly from any other type of injuries normally encountered in Germany. Penetrating injuries due to gunshot or blast injuries could be predominant related to the scenario [3, 8]. *These types of injuries had to be managed during the Wurzburg, Ansbach and the Munich incident. They also were predominant in Paris and Brussles. Major blunt trauma occurred during the Berlin Christmas market assault, while a truck drove in a crowd of people.* Therefore, early identification of causalities with uncontrolled bleeding and their prioritised transportation into a hospital nearby, where emergency surgical care is delivered without delay, is the major challenge for preclinical and in-hospital treatment provision.

Psychosocial crisis management is a very important issue within the respond to terrorist attacks [2, 9]. An evaluation of the governmental psychosocial support of German victims and their relatives from 46 different terror attacks abroad within the last 15 years indicates specific distinctions in the psychosocial needs and demands of people affected by terror attacks in contrast to other mass casualty incidents [10–12].

## Methods

On behalf of the German Federal Ministry of the Interior and the German Federal Ministry of Health and under the direction of the German Federal Office of Civil Protection and Disaster Assistance, a panel of experts was established to define the most important lessons learned from the recent terror attacks in Europe. The expert group consisted of leading representatives of authorities, organisations and institutions directly involved in the management of active killing events.

The definition of the lessons learned for civil rescue missions during terror attacks was based on three expert meetings. These meetings were all held in an identical and structured methodical way. At the beginning, there was the task definition, followed by the elaboration of key points, which were then consented by the expert group. No standardized or broadband Delphi method was used for the definition of the lessons learned. Most important reason for that was the lack of anonymity among the experts which is one dedicated characteristic of the Delphi method.

The general pattern of the of the lessons learned definition was built on three levels: (1) formulation of the general mission goals, (2) definition of the strategic features necessary for obtaining these goals and (3) determination of the tactical requirements for successful realization.

The results of the whole process are an expert opinion and should be interpreted as that.

## Results

### of common goals, mission strategies and tactics

First of all, the formulation of common goals for rescue missions during active killing events is important. This applies to the mission planning and execution phase, as it allows the different rescue forces to understand the key aspects of the mission and adapt their own strategies accordingly. By having common goals pre-identified, communication during a mission can be goal and information oriented, potential conflicts can be resolved beforehand, and trust and mutual respect can be promoted. Having a common goal and common sense is the key to a successful mission outcome. To obtain these goals, mission strategies and tactical requirements have to be developed and defined in advance. The presented bullet point recommendations show the basic and most important results of the ongoing evaluation process for the Federal Republic of Germany.

#### Definition of the common goals

1. The safety and security of the rescue forces.
2. The survival of the greatest possible number of casualties.

#### To obtain these goals, special strategic features during life-threatening mass casualty incidents are as follows:

1. Necessity of immediate defusing of threat situation (strategic incident priorities: stop the killing, stop the dying) [6, 7, 13].
2. Early identification and prioritised transportation of casualties with life-threatening non-controllable bleeding [6, 7, 13].
3. Shortest possible on-scene time: “Clear up the scene immediately”.

#### Tactical requirements during life-threatening mass casualty incidents: key points

1. Development of defined and identical medical and tactical standards.
  Prehospital casualty care.
    Establishment of command and control structures [6, 7, 13, 14].
    Police and emergency medical service coordinate arrangement of the area and management of the scene. Definition and communication of the unsafe zone (hot zone), definition and communication of casualty collection points, triage area (warm zone/semi-safe zone) and a safe treatment area (cold zone/safe zone) [6, 7, 13, 14].
    Rapid initial triage and identification of the most serious injured patients (“Priority 1” patients) [6, 7, 13, 14].
    Immediate treatment of potentially survivable life-threatening problems (exsanguination, airway obstruction and tension pneumothorax) by initial resuscitation measures and identification of casualties with life-threatening non-controllable bleeding [6, 7, 13, 14].
    Acceleration of the evacuation flow towards more secure areas and the casualty collection points [6, 7, 13, 14].
    Prioritised and timely transport of “Priority 1” patients to nearby hospitals.
    Adapted medical treatment on the basis of Tactical Combat Casualty Care (TCCC) [13, 15].
    Consider and prepare for special groups of patients (e.g. children) [4].
    Consider and prepare for special threats (e.g. biological and chemical weapons/ CBRN) [4].
  In-hospital casualty care.
    Increase treatment capacity by applying hospital emergency plans [16].
    Accept an initial phase of uncertainty with a lack of resources (personal and material) with the appearance of bleeding casualties [16].
    Organisation of emergency surgical care following the algorithm: Categorisation–Prioritization–Disposition–Realization [17, 18].
      Fast triage and identification of “Priority 1” patients (categorization).
      Prioritization of patients with non-controlled bleeding and hemodynamic instability (in shock) for immediate surgery.
      Disposition of essential, not decelerating diagnostic.
      Realization of surgical treatment aiming to enable survival in as many as possible casualties.
    During the initial phase or in a mass casualty event due to a terroristic incident reduce surgical care to tactical abbreviated surgery care (TASC) principles: keep it short and simple (KISS) only aiming to survival [17–19].
    Increase stock of material needed for the treatment of penetrating injuries to thorax and abdomen [4].
    Increase endurance through human resource planning.
    Consider and prepare for special groups of patients (e.g. children) [4].
    Consider and prepare for special threats (e.g. biological and chemical weapons/ CBRN) [4].
2. Resource management (human resource and material).
  Early alert of regional and national rescue forces and hospitals.
  Creation of human resource reserves—if necessary over days.
  Stockpiling of personal protective equipment.
  Stockpiling of tourniquets and other material needed for the treatment of massive bleeding as from penetrating injuries.
  Logistics of delivery of material to incident scene.
3. Psychosocial crisis management
  Structural integration of psychosocial emergency care (PSNV) into hazard avoidance tactics (including crisis management group).
  Professional acute-, mid- and long-term psychosocial support offers for victims.
  Qualified preventative and follow-up care for rescue team members and their families.
  Use of executive personnel of the psychosocial care team.
  Consideration of socio scientific research.
4. Communication.
  Establishment of a communication plan between the law enforcement and all other rescue forces.
  Prioritised and early communication between the police and other rescue forces (especially between the headquarter/dispatch center of the police forces and the emergency medical services).
  Early and frequent communication between the different commanders in chief of the different rescue services involved in the mission.
  Uniform communication tactics.
  Identification and definition of fixed communication partners during mission.
5. Casualty identification and support for relatives.
  High priority of casualty identification.
  High priority of information relay and support for relatives.
6. Combined education, training and practice.
  Preclinical training of real life scenarios with all involved forces and hospitals.
  In-hospital exercises including the evaluation of resources needed for surgical care.
  Development of courses and manuals for preclinical decision-making (e.g. TCCC) and promotion of in-hospital decision-making and emergency surgical care training (terror and disaster surgical care, TDSC) [15, 18].
  Supporting exercises by public funding.

## Discussion

The definition of the common goals, the identification of the special strategic features and the tactical key points summarise the most important and basic results of the evaluation process on a national level of the terrorist attacks for the Federal Republic of Germany. These points have been given highest implementation priority in view of hazard prevention tactics within Germany. For example, we picked communication as a specific bullet point, as it was one of the most critical issues in the incident reports of the evaluated rescue missions in Germany. Naturally, the process does not end with the identification of weaknesses, the definition of goals, strategies and tactics. The focus has now shifted to intensive work and research about possible solutions for the identified deficits and implementation strategies of such solutions. To obtain these objectives, the German Federal Office of Civil Protection and Disaster Assistance conducted several conferences in 2018 with thematic priorities to formulate conclusions and recommendations. These have been finished and partly published in 2019 [16, 20].

Other European countries also have followed dedicated pattern of evaluation and publication of the results of the management of the terrorist attacks they have experienced [1–4]. Hirsch et al. deserve a special mention for the publication of the first lessons learned directly after the terror attacks in France [1]. A follow-up publication identifies the weaknesses and describes the current implementation stage of the found solutions [4].

The authors identified 12 bullet points [4]:

- insufficient expertise on war weapons
- prehospital damage control
- children as victims of ballistic trauma
- chemical weapons
- health-care facilities as target
- secure intervention of medical responders under fire
- triage at scene and at the arrival of the hospital
- terrorist attacks in areas with insufficient medical resources
- identification of victims
- care of the psychological victims
- international medical network on terrorist attacks
- Unexpected terrorist innovation.

Most of the 12 identified weaknesses are similar to our findings and conclusions. The cooperation on an international level should be expanded to find common solutions.

To improve medical treatment of war weapon caused injuries and to teach the principles of Tactical Abbreviated Surgical Care, the German Society of Trauma and Orthopedics in close cooperation with the German military, has recently developed the specific course concept “Terror and Disaster Surgical Care” (TDSC). The course is offered and trained on a national level [18] and was recently matched with the principles of Advanced Trauma Life Support [21].

The aspects of the tactical emergency medicine were described by the Service Medical du RAID (Research, Assistance, Intervention, Deterrence), the French national police counter-terrorism team, which was engaged during the Bataclan terrorist attack [22]. The authors described the zoning, the medical treatment “under fire” with the most important aspects of initial triage and resuscitation. The tactical physicians performed resuscitation within the combat zone and moved invalid casualties towards more secured areas. The conventional rescue teams had to stay outside the danger zone until complete threat suppression and finishing the mine-clearing operations [22]. The most important difference to the German System is the lack of tactical physicians within Germany. This brings high priority to the cooperation of the police forces and the medical rescue forces as the police forces have to act as evacuation flow accelerators in the combat zone. We have addressed these aspects in the strategic features and the tactical features as well.

After a terrorist attack in Wurzburg, Germany, Wurmb et al. [23] defined quality indicators to better evaluate rescue missions für mass killing incidents. These quality indicators may serve as a basis for further research within this field.

Brandrud and co-workers did an evaluation of the Utoya terrorist attack in 2011. The authors identified four essential elements for successful management of mass killing incidents [22]. (1) Structure and competence based on continuous planning, training and learning, (2) leadership based on knowledge trust and data collection, (3) empowerment through multiprofessional networks and (4) ability to improvise based on structure and competence.

The prerogative to fulfil these bullet points is a meticulous and careful evaluation process to identify weaknesses and to formulate solutions and plans. This is the basic goal of the federal-conducted ongoing evaluation process in Germany.

## Limitations

The report does not include recommendations for chemcial–biological or radio-nuclear threats (CBRN). Furthermore, it does not address special subgroups such as paediatric patients or burns. These issues will be handled during the ongoing evaluation process.

At that stage, the report does not give final recommendations on how to operationalize the key points. To formulate guidelines, to develop and implement teaching curricula and to perform common drills will be the challenge of the next years.

## Conclusion

Active killing events pose the highest demands to the partaking rescue forces. It is essential to control the source of threat immediately, whilst simultaneously rescuing the greatest possible number of casualties and trying to protect the lives of all rescue team members. To obtain these objectives, it is vital to develop, implement and train collective and realistic concepts and guidelines, which can be applied effectively for emergency response to terrorist attacks and other active killing events.
